# The remission phase in adolescents and young adults with newly diagnosed type 1 diabetes mellitus: prevalence, predicting factors and glycemic control during follow-up

**DOI:** 10.20945/2359-3997000000456

**Published:** 2022-03-23

**Authors:** Meriem Yazidi, Sana Mahjoubi, Ibtissem Oueslati, Fatma Chaker, Melika Chihaoui

**Affiliations:** 1 University of Tunis El Manar Faculty of Medicine of Tunis Department of Endocrinology Tunis Tunisia Department of Endocrinology, University of Tunis El Manar, Faculty of Medicine of Tunis, La Rabta Hospital, Tunis, Tunisia

**Keywords:** Type 1 diabetes, remission, HbA1c, adults, adolescents

## Abstract

**Objective:**

There is little data about the remission phase in adolescents and young adults with newly diagnosed type 1 diabetes mellitus (T1D). The aims of this study were to determine the prevalence of remission and its predicting factors among adolescents and young adults with newly diagnosed T1D and to assess the association between remission and long-term glycemic control in this population.

**Subjects and methods:**

This is a longitudinal and retrospective study including 128 type 1 diabetic patients aged between 12 and 30 years at diabetes onset. Clinical, biological and therapeutic features were collected at diagnosis and for 5 years after diagnosis. Remission was defined by an HbA1c < 6.5% with a daily insulin dose < 0.5 IU/kg/day.

**Results:**

Twenty-three patients (18%) experienced a remission. The peak of remission prevalence was at 6 months after diabetes diagnosis. An insulin dose at discharge <0.8 IU/kg/day was independently associated with remission (p=0.03, adjusted OR [CI 95%] = 0.2 [0.1-0.9]). A low socioeconomic level was independently associated with non remission (p=0.02, adjusted OR [CI 95%] = 4.3 [1.3-14.3]). HbA1c was significantly lower during the first five years of follow-up in remitters. The daily insulin dose was significantly lower during the first four years of follow-up in remitters.

**Conclusions:**

Occurrence of remission in adolescents and young adults with newly diagnosed T1D is associated with better glycemic control and lower insulin requirements during the first 5 years of follow-up. A lower initial dose of insulin was associated with a higher percentage of remission.

## INTRODUCTION

Some patients with newly diagnosed type 1 diabetes (T1D) experience, after starting insulin treatment, a period of clinical and metabolic remission commonly known as the “honeymoon phase”. This phase is characterized by a decrease in insulin requirements and an optimal glycemic control. It is due to a partial and transient improvement in the function of β-cells ( 1 ). It usually occurs within 12 months, sometimes later, after the diagnosis and lasts between 7 and 12 months ( 2 ). The reported prevalence of the remission phase varies considerably due to the different definitions of remission itself. This phase would be associated with better long-term glycemic control and a decreased risk of chronic complications ( 3 ). Several factors, such as age, gender, diabetes-related autoantibodies and metabolic parameters at presentation, have been suggested as influencing the incidence of remission especially in a pediatric population ( 4 ). Less is known about adolescents and adults with recent-onset T1D.

The aims of the present study were to determine the prevalence of remission and its predicting factors among adolescents and young adults with newly diagnosed T1D and to assess the association between remission and long-term glycemic control in this population.

## SUBJECTS AND METHODS

This is a longitudinal and retrospective study including 128 patients with newly diagnosed T1D admitted to the endocrinology department of the Rabta Hospital in Tunis, Tunisia, between January 1998 and December 2011. Only patients aged between 12 and 30 years at diabetes onset and followed for at least one year were included.

The diagnosis of T1D was based on a body of clinical and biological arguments: ketoacidosis at diabetes diagnosis, weight loss, insulin requirement, positive islet cell antibodies (ICA) or glutamic acid decarboxylase antibodies (GADA).

### Data collection and definition of the studied parameters

Data were retrospectively collected from the medical records from the diagnosis until the fifth year of follow-up for the following parameters: the socio-demographic (age, gender and socioeconomic status), clinical (diseases associated with diabetes, weight and height at diagnosis), biological (blood glucose value, ketonuria, pH, serum bicarbonate and HbA1c at diagnosis) and therapeutic (insulin regimen and insulin dose at discharge).

Remission was defined by an HbA1c < 6.5% with a daily insulin dose < 0.5 IU/kg/day. Complete remission was defined by an HbA1c < 6.5% without insulin.

Patients were classified into two age groups: adolescents (12-18 years) and young adults (19-30 years).

Indigent patients receiving free medical care were classified as having a low socioeconomic status.

Diabetic ketoacidosis (DKA) was diagnosed when serum bicarbonate was < 15 mmol/L or pH < 7.3 with ketonuria and hyperglycemia.

The weight status of adult subjects was determined according to the body mass index (BMI) WHO classification ( 5 ). The weight status of adolescents was determined using age- and sex-adjusted body mass index curves. Patients were classified into three categories according to their BMI: normal-weight, underweight and overweight subjects.

The rate of remission was assessed at 3 months, 6 months, 1 year, 2 years, 3 years, 4 years and 5 years after diagnosis. Patients who went into remission (remitters) were compared to those who did not experience remission (non-remitters) according to their socio-demographic, clinical, biological and therapeutic characteristics. Glycemic control during follow-up was assessed in each group at 3 and 6 months and 1, 2, 3, 4 and 5 years after diagnosis, based on the mean values of HbA1c. The daily insulin dose during follow-up was also assessed in both groups.

HbA1c was measured by the high-performance liquid chromatography (HPLC) method.

The study was approved by the hospital's ethical committee.

### Statistical analysis

Statistical analyses were performed with SPSS software for Windows, version 20 (SPSS Inc., Chicago, IL). Data were expressed as means ± SD for quantitative variables and percentages for qualitative variables. The Student's *t* test was used to compare means between independent groups. Pearson's chi-square and Fisher's exact tests were used to compare proportions.

Receiver operating characteristic (ROC) curves were depicted, allowing determination of optimal cutoff values for age, initial blood glucose level, HbA1c value and insulin dose at discharge.

A stepwise binary logistic regression model was applied to determine the variables independently associated with remission. All the factors with a p-value <0.2 on the univariate analysis were included in the multivariate logistic regression model. A p-value <0.05 based on two-tailed calculations was considered significant.

## RESULTS

The mean age of the patients at diagnosis was 21.4 ± 4.7 years. There were 77 males and 51 females. Eighty-four patients (65.6%) were adults and 44 (34.4%) were adolescents. Six patients (4.7%) had an associated autoimmune disease (2 patients had Hashimoto's thyroiditis and 4 patients had coeliac disease). At discharge, 117 patients (91.4%) were on an intensive insulin regimen consisting of three injections of regular insulin daily with one or two injections of NPH insulin (basal-bolus regimen). The others (11 patients) were on an insulin regimen with two injections per day. During follow-up, 21% of patients were on insulin analogues.

### Remission prevalence during follow-up

**Figure 1:**
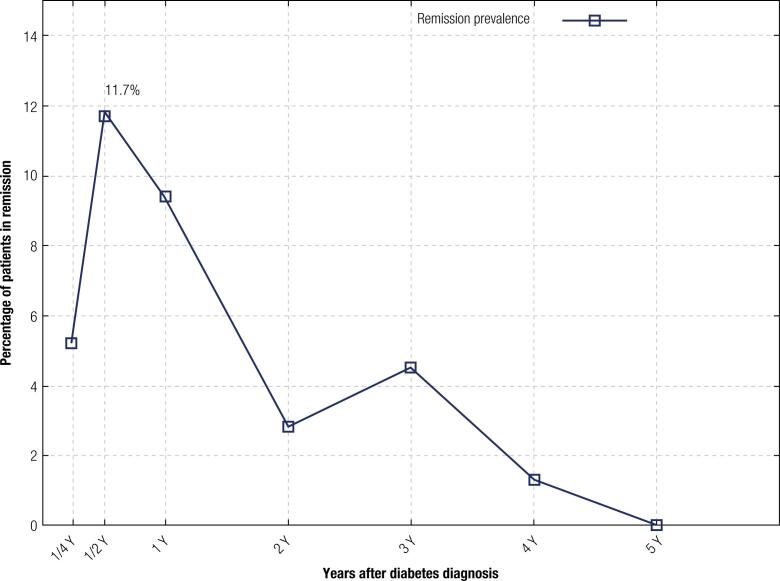
Remission prevalence during follow-up

During the follow-up period, 23 patients (18%) experienced a remission. The peak of remission prevalence was at 6 months after diabetes diagnosis ( Figure 1 ). Two patients (1%) had a complete remission with persistence of an HbA1c of less than 6.5% despite discontinuation of insulin therapy. This complete remission occurred 6 months after the diabetes diagnosis for the first patient and 12 months after diabetes diagnosis for the second patient. Its mean duration was 3 months for the first patient and 6 months for the second patient.

### Factors associated with remission

The univariate and multivariate analyses of the baseline parameters according to the occurrence of a remission are shown in Table 1 . After multivariate analysis, a low insulin dose was an independent factor for remission, and low socioeconomic status was an independent factor for non-remission.

**Table 1:** Univariate and multivariate analyses of the baseline parameters according to the occurrence of a remission

	Univariate analysis				Multivariate analysis	
	Remitters (n=23)	Non remitters (n=105)	p value	OR [CI 95%]	p value	Adjusted OR [CI 95%]
Age < 21 years (%)	43.5	52.4	0.43	0.7 [0.3-1.7]		
Adolescents (%)	17.4	38.1	**0.05**	**0.3 [0.1-1.0]**	0.28	0.5 [0.1-1.7]
Male gender (%)	65.2	59.0	0.58	1.3 [0.5-3.3]		
Low socioeconomic status (%)	13.6	47.6	**0.03**	**0.2 [0.0-0.6]**	0.03	0.2 [0.1-0.9]
Associated autoimmune disease (%)	10.0	4.0	0.26	2.6 [0.4-15.7]		
Underweight subjects (%)	13.0	26.7	0.16	0.4 [0.1-1.5]	0.64	0.7 [0.2-3.0]
Ketoacidosis at diagnosis (%)	17.4	18.1	0.93	0.9 [0.3-3.1]		
Initial blood glucose value < 3.7 g/l (%)	65.2	46.5	0.11	2.1 [0.8-5.5]	0.38	1.6 [0.5-4.7]
HbA1c value at diagnosis < 14.2% (%)	45.5	51.5	0.73	0.8 [0.2-3.1]		
Insulin dose at discharge < 0.8 IU/Kg/day (%)	82.6	41.9	**<0.001**	**6.6 [2.1-20.7]**	**0.02**	**4.3 [1.3-14.3]**

OR: odds ratio; CI: confidence interval.

### Glycemic control and daily insulin dose during follow-up

Glycemic control and a daily insulin dose during follow-up in remitter and non-remitter patients are shown in Table 2 . HbA1c was significantly lower during the first five years of follow-up in remitters. Daily insulin dose was significantly lower during the first four years of follow-up in remitters.

**Table 2:** Glycemic control and daily insulin dose during follow-up in remitter and non remitter patients

	Remitters	Non remitters	p
HbA1c at 3 months (%)	6.5 ± 1.9	8.5 ± 2.5	**0.02**
Insulin dose at 3 months (IU/kg/day)	0.42 ± 0.2	0.82 ± 0.3	**<0.001**
HbA1c at 6 months (%)	5.8 ± 0.6	7.6 ± 2.6	**<0.001**
Insulin dose at 6 months (IU/kg/day)	0.35 ± 0.2	0.79 ± 0.3	**<0.001**
HbA1c at 1 year (%)	7.1 ± 1.6	8.7 ± 2.4	**0.004**
Insulin dose at 1 year (IU/kg/day)	0.37 ± 0.2	0.75 ± 0.3	**<0.001**
HbA1c at 2 years (%)	7.8 ± 1.8	9.7 ± 2.5	**0.004**
Insulin dose at 2 years (IU/kg/day)	0.48 ± 0.2	0.73 ± 0.3	**0.003**
HbA1c at 3 years (%)	8.2 ± 1.9	9.8 ± 2.4	**0.032**
Insulin dose at 3 years (IU/kg/day)	0.46 ± 0.1	0.78 ± 0.3	**0.001**
HbA1c at 4 years (%)	7.8 ± 1.8	10.1 ± 2.6	**0.008**
Insulin dose at 4 years (IU/kg/day)	0.55 ± 0.2	0.82 ± 0.2	**0.001**
HbA1c at 5 years (%)	8.3 ± 1.2	9.6 ± 2.5	**0.018**
Insulin dose at 5 years (IU/kg/day)	0.62 ± 0.2	0.78 ± 0.2	0.129

## DISCUSSION

There is currently little data regarding the remission phase in adolescents and young adults with newly diagnosed T1D. In the present study the prevalence of remission defined by an HbA1c < 6.5% and an insulin dose < 0.5 IU/kg/day in this population was 18.5%. A low insulin dose was an independent factor for remission, and low socioeconomic status was an independent factor for non-remission. Remission was associated with better glycemic control and a lower daily insulin requirement during follow-up. The main limitation of this study is related to its retrospective design. Some factors that may be involved in the onset of remission in type 1 diabetics have not been studied, such as symptoms at presentation (polydipsia, polyuria and weight loss), ICA, GADA and C peptide level at diagnosis.

### Remission frequency

The frequency of the remission phase in type 1 diabetes depends directly on the definition used and therefore varies widely from one study to another. In addition, age, ethnicity and insulin treatment regimen of the populations studied differ across studies, limiting comparisons and interpretation of their results. There is little data on adolescents and adults ( 2 , 3 , 6 – 8 ). The prevalence of remission reported by different studies in T1D adolescents and adult patients over the last two decades are summarized in Table 3 . The prevalence found in our study is much lower than that reported in the other studies. In the pediatric population, the prevalence of remission is between 11 and 80% ( 9 ). A new definition for partial remission in children and adolescents with T1D was proposed by Mortensen et al. in 2009: A1C (%) + [4 × insulin dose (units per kilogram per 24 h)] ≤9. It is well correlated with the residual β-cell function estimated by the level of stimulated C peptide ( 10 ).

**Table 3:** Studies of remission prevalence in adolescents and adults with type 1 diabetes

Study (Country)	Patients characteristics	Diagnostic criteria of remission	Remission prevalence
Schölin et al. 1999 (Sweden) ( 2 )	62 new-onset T1D patients aged between 16 and 50 years	HbA1c ≤ 6.5% and an insulin dose ≤ 0.4 IU/kg/day for at least one month	61%
Scholin et al. 2004 (Sweden) ( 6 )	362 new-onset T1D patients aged between 15 and 35 years	HbA1c lower than the upper laboratory limit and an insulin dose < 0.3 IU/Kg/day	43%
Scholin et al. 2011 (Sweden) ( 7 )	78 new-onset T1D patients aged between 15 and 34 years	HbA1c within the normal range and an insulin dose < 0.3 IU/kg/day	45%
Pilacinski et al. 2012 Poland ( 8 )	149 new-onset T1D patients aged between 16 and 35 years	HbA1c value <7.0% with an insulin dose <0.3 IU/kg/day and a random serum C-peptide concentration > 0.5 ng/mL	46%
Niedzwiecki et al. 2015 Poland ( 3 )	90 new-onset T1D patients aged between 18 and 35 years on functional insulin therapy	HbA1c < 6.5%, daily insulin requirement < 0.3 IU/kg/day and serum C peptide concentration >0.5 ng/mL	66%
Present study	128 new-onset T1D patients aged between 12 and 30 years	HbA1c < 6.5% and an insulin dose < 0.5 IU/kg/day	18.5%

T1D: Type 1 diabetic.

As in our study, the peak prevalence of remission usually occurs 3 to 6 months after the start of insulin therapy ( 1 ). The time of the onset of remission was earlier in some series by up to 14 days or less ( 11 , 12 ).

### Factors associated with remission

A number of factors have been associated with the frequency of remission in T1D. A younger age at the time of diagnosis would be associated with a more aggressive autoimmune process and a faster destruction of β cells, resulting in a lower chance of going into remission ( 4 , 13 – 15 ). In our study, the frequency of remission was lower in adolescents compared to adults in univariate analysis. In addition to age, this finding can be explained by a more pronounced state of insulin resistance in adolescents due to an increase in the growth hormone and sex hormone levels during puberty ( 16 ). The association between gender and remission is very controversial ( 17 ). In this study, no significant influence of gender on remission was demonstrated. The frequency of remission was significantly lower in patients with poor socioeconomic status. This association persisted even after multivariate analysis. There are no data addressing the relationship between the socioeconomic level and the remission rate in type 1 diabetic patients. Carter et al, showed that ethnicity and social deprivation independently influence metabolic control in children with type 1 diabetes ( 18 ). A lower socioeconomic level could be associated with delayed diagnosis and poorer management of diabetes, resulting in a lower function in residual beta cells and a lower prevalence of remission.

Ketoacidosis at diagnosis was associated with a lower frequency of remission ( 2 , 6 , 14 , 15 , 19 , 20 ). Ketoacidosis reflects a severe insulin deficiency and therefore impaired function in residual beta cells ( 21 ). The lack of relationship between ketoacidosis and remission in our study could be explained by an underestimation of the frequency of acidosis since arterial blood gas analyses were not systematically performed in all patients at the time of diagnosis.

Several studies have demonstrated that the prevalence of adolescent obesity has risen during the past 30 years in type 1 diabetic patients ( 22 ). The association between body weight at the diagnosis of diabetes and remission is also controversial. Many studies have not found an association ( 2 , 23 , 24 ). Others, however, have observed a higher prevalence of the “honeymoon phase” in patients with higher BMI at diagnosis ( 6 , 7 , 25 ). This finding can be explained by the fact that a higher BMI at the time of diagnosis would indicate a less severe state of insulinopenia. A positive correlation between the BMI at diagnosis and the level of C peptide has been demonstrated in type 1 diabetic patients ( 26 ).

Glucose and HbA1c levels at diagnosis were not associated with remission in our study. Other studies have shown that the initial value of HbA1c was significantly associated with the rate of remission ( 8 , 27 ). HbA1c reflects chronic hyperglycemia during the past three months. This hyperglycemia has a gluco-toxic effect on Langerhans beta cells, resulting in a reduction in insulin secretion ( 28 ). On the other hand, hyperglycemia causes an increase in the level of surface antigens exposed to autoimmune cells. The consequence is a more accelerated destruction of beta cells.

In our study, the insulin dose at discharge was lower in subjects who went into remission. This significant difference persisted after multivariate analysis. This finding is consistent with the results of other studies ( 6 , 29 ). The exogenous insulin requirement reflects the state of the pancreatic beta cells: the higher these requirements are, the lower the residual beta cells function is, hence a lower chance of going into remission.

### Long term glycemic control in remitter patients

Few studies have investigated the long-term influence of the occurrence of a remission in adolescents and adults with T1D. Better glycemic control during follow up has been reported in type 1 diabetic patients who experienced a remission period ( 3 , 25 , 30 ). In our study, remission was associated with lower HbA1c and lower daily insulin requirements during the 5 first years of diabetes diagnosis. These results may be explained by the persistent function in residual beta cells if remission occurs during the natural history of T1D. This is important to consider since it has been shown that good glycemic control during the first years of a T1D diagnosis had a persistent effect and is involved on the time of onset of microangiopathies ( 31 , 32 ). Niedzwiecki and cols., showed a reduction in the risk of chronic microvascular complications after 7 years of follow-up in type 1 diabetic patients who experienced a remission period. Microangiopathic complications were six times more common in non-remitter patients ( 3 ).

Through this study, we highlight the importance of the occurrence of remission in adolescents and young adults with T1D. The management of diabetes is easier and metabolic control is easily obtained with low doses of insulin. Interventional studies aimed at inducing and prolonging the remission period in type 1 diabetics should be promoted. Immunosuppressive therapies have demonstrated their effectiveness, but their side effects outweigh their benefits ( 33 ). Other therapeutic approaches using less harmful molecules are needed.
